# Risk factors and prognostic implications of surgery-related strokes following resection of high-grade glioma

**DOI:** 10.1038/s41598-022-27127-5

**Published:** 2022-12-30

**Authors:** Assaf Berger, Garry Gali Tzarfati, Marga Serafimova, Pablo Valdes, Aaron Meller, Akiva Korn, Naomi Kahana Levy, Daniel Aviram, Zvi Ram, Rachel Grossman

**Affiliations:** 1 Department of Neurosurgery, Tel Aviv Medical Center, 6 Weizmann St, 6423906 Tel Aviv, Israel; 2 Division of Anesthesiology, Tel-Aviv University, Tel-Aviv, Israel; 3 Sackler Faculty of Medicine, Tel-Aviv University, Tel-Aviv, Israel; 4 Department of Neurosurgery, NYU Langone Medical Center, New York University, 530 First Avenue, New York, NY 10016 USA

**Keywords:** Cancer, CNS cancer, Stroke

## Abstract

Surgery-related strokes are an important cause of morbidity following resection of high-grade glioma (HGG). We explored the incidence, risk factors and clinical consequences of intra-operative ischemic strokes in surgeries for resection of HGG. We retrospectively followed a cohort of 239 patients who underwent surgical resection of HGG between 2013 and 2017. Tumor types included both isocitrate dehydrogenase (IDH) wildtype glioblastoma and IDH-mutant WHO grade 4 astrocytoma. We analyzed pre- and post-operative demographic, clinical, radiological, anesthesiology and intraoperative neurophysiology data, including overall survival and functional outcomes. Acute ischemic strokes were seen on postoperative diffusion-weighted imaging (DWI) in 30 patients (12.5%), 13 of whom (43%) developed new neurological deficits. Infarcts were more common in insular (23%, p = 0.019) and temporal surgeries (57%, p = 0.01). Immediately after surgery, 35% of patients without infarcts and 57% of those with infarcts experienced motor deficits (p = 0.022). Six months later, rates of motor deficits decreased to 25% in the non-infarcts group and 37% in the infarcts group (p = 0.023 and 0.105, respectively) with a significantly lower Karnofsky-Performance Score (KPS, p = 0.001). Intra-operative language decline in awake procedures was a significant indicator of the occurrence of intra-operative stroke (p = 0.029). In conclusion, intraoperative ischemic events are more common in insular and temporal surgeries for resection of HGG and their intra-operative detection is limited. These strokes can impair motor and speech functions as well as patients’ performance status.

## Introduction

Maximal safe resection of high-grade glioma (HGG) has been linked to improved overall and progression-free survival^1,2^. Surgery-related new or deteriorating neurological deficits, on the other hand, have been associated with impaired quality of life and even decreased survival^3,4^. A significant proportion of these complications result from intra-operative ischemic events, which occur in 6.2 to 80%, depending on patients populations and stroke diagnostic definitions^5–9^.

Previous studies have evaluated mixed cohorts of both high- and low- grade tumors, limiting the ability to draw specific conclusions for these two distinctly different populations. Moreover, most studies described clinical complications but did not follow these for the long term^6,10–12^. This study is a continuum to our published results on low-grade glioma population^13^. We now focused on evaluating the incidence of intra-operative ischemic events and their risk factors in patients undergoing resection for HGG. In addition, we examined possible intraoperative factors suggestive of evolving stroke.

## Results

### Clinical and demographic data

Between the January 2013 to December 2017, 348 patients underwent surgical resection or biopsy for newly diagnosed and recurrent HGG at the Tel Aviv Medical center. Patients that were excluded from our study were those who underwent biopsy for the diagnosis of HGG (n = 39), had missing pre-and postoperative MRI data (n = 19), or had no detailed admission, surgery, and discharge reports (n = 51). Full datasets, including long-term clinical outcomes were available for 239 patients who underwent resection, and they comprised our study cohort. The mean age at surgery was 58 ± 14 years, and 150 (63%) were males. Preoperative new-onset manifestations included headache (n = 96, 40%), motor deficits (n = 92, 38.5%), speech deficits (n = 69, 28.5%), and seizures (n = 80, 33.5%). The median and range of preoperative KPS and MRS were 80 (30–100) and 2 (0–5), respectively. Tumors were located in the frontal (n = 109, 46%), temporal (n = 85, 36%), parietal (n = 69, 29%), and insular lobes (n = 26, 11%). Average preoperative tumor volume was 33 ± 26 ml, and EOR was 96 ± 7%. Eighty-six patients (36%) had recurrent surgeries for tumor progression, of which 63 (73%) also had radiotherapy prior to their most recent studied surgery. Awake operations were performed in 73 (31%) of the cases. All HGG cases had pathological diagnoses of isocitrate dehydrogenase (IDH) wildtype glioblastoma (88%) or IDH-mutant World Health Organization (WHO) grade 4 astrocytoma (12%), according to the 2021 WHO classification^14^.

### Stroke incidence and risk factors

Acute ischemic strokes were evident in proximity to the resection cavity on immediate postoperative MRI in 30 patients (12.5%), and 13 of them (43%) developed new neurological deficits resulting from the ischemic event. The study groups were compared in a univariate analysis in Table 1. Infarcts were more common in patients undergoing surgery for tumors located in the insula (23%) and temporal lobe (57%), p = 0.019 and p = 0.01, respectively. In addition, a higher pre-operative level of platelets was associated with a higher risk of stroke (p = 0.034). In this study we found no significant association between recurrent surgeries and the risk of stroke (p = 0.746). However, after a multivariate analysis for potential confounders (Table 2), surgery for insular HGG had the strongest association with postoperative infarcts (multivariate analysis: odds ratio [OR] 2.41, 95% confidence interval, [CI] 1.02–7.73, p = 0.045, followed by temporal lesions (OR 2.81, 95% confidence interval, [CI] 1.07–5.39, p = 0.033). Platelet levels did not show a significant association with stroke on the multi-variate analysis (p = 0.057). Most (16/30; 53%) of the strokes involved segments of the middle cerebral artery (MCA) territory, of which 7/16 (44%) were insular and involved small perforating arteries. Twelve of the infarcts (40%) involved territories of the posterior cerebral artery and 2 (7%) of the anterior cerebral artery. Average infarct size measured 13.2 ± 13.9 cc.

**Table 1:** Clinical and demographic data of the entire study population, including comparisons between patients with and without intra-operative strokes.

	Entire study population (n = 239)	No Infarct n = 209 (87.5%)	Infarct n = 30 (12.5%)	Sig.
Age	59 ± 14	58 ± 14	61 ± 12	0.193
Sex (male, %)	150 (62.8%)	130 (62.2%)	20 (66.7%)	0.636
BMI (Kg/m^2^)	26.6 ± 3.9	26.6 ± 3.9	26.1 ± 3.2	0.483
Pre-op KPS ≥ 70, %	200 (84%)	175 (84.1%)	25 (83.3%)	0.911
Pre-op MRS ≤ 3, %	217 (91.2%)	191 (91.8%)	26 (86.7%)	0.352
Ever smoking, %	28 (11.7%)	26 (12.4%)	2 (6.7%)	0.358
Diabetes mellitus, %	34 (14.2%)	28 (13.4%)	6 (20%)	0.333
Stroke, %	4 (1.7%)	3 (1.4%)	1 (3.3%)	0.449
Ischemic Heart Disease, %	24 (10%)	19 (9.1%)	5 (16.7%)	0.197
Deep Venous Thrombosis, %	6 (2.5%)	5 (2.4%)	1 (3.3%)	0.758
Hypertension, %	85 (35.6%)	71 (34.1%)	12 (40%)	0.187
Dyslipidemia, %	71 (29.7%)	59 (28.2%)	11 (39.3%)	0.238
Other malignancy, %	27 (11.3%)	21 (10%)	6 (20%)	0.107
Pre-op serum Hb, g/dL	13.57 ± 1.75	13.54 ± 1.75	13.63 ± 1.47	0.792
Pre-op platelets (*10^3^)	216 ± 70	217 ± 73	249 ± 85	**0.034**
Recurrent, %	86 (36%)	76 (36.4%)	10 (33.3%)	0.746
Previous radiation, %	69 (28.9%)	57 (27.3%)	12 (40.0%)	0.150
Awake, %	73 (31%)	68 (32.5%)	5 (16.7%)	0.078
Tumor enhancement, %	237 (99.2%)	207 (99.0%)	30 (100%)	0.591
Tumor volume pre-op, cc	32.76 ± 24.52	33.05 ± 25.65	33.87 ± 25.83	0.881
EOR, %	96.3% ± 7.2	96.10% ± 7.64	95.9% ± 6.26	0.907
P53 + , %	72 (41.9%)	63 (41.4%)	9 (45%)	0.762
IDH 1 + , %	22 (11.9%)	22 (13.5%)	0	0.066
Frontal, %	109 (45.6%)	99 (47.4%)	10 (33.3%)	0.149
Insular, %	26 (11%)	19 (9.1%)	7 (23.3%)	**0.019**
Temporal, %	85 (35.6%)	68 (32.5%)	16 (56.7%)	**0.01**
Parietal, %	69 (28.9%)	63 (30.1%)	6 (20.0%)	0.252
Occipital, %	25 (10.5%)	20 (9.6%)	5 (16.7%)	0.235
Gliadel, %	28 (11.7%)	27 (12.9%)	1 (3.3%)	0.127

Significant values are in bold.

*BMI body mass index, *KPS* Karnofsky performance status, *MRS* modified rankin scale, *Hb* hemoglobin, *EOR* extent of resection, *IDH* isocitrate dehydrogenase 1.

**Table 2:** Multivariate logistic regression analysis for estimating risk factors for intra-operative stroke.

	Infarcts OR (95% CI)	Sig.
**Risk**		
Insular lesion	2.81 (1.02–7.73)	0.045
Temporal lesion	2.41 (1.07–5.39)	0.033
Platelets	1.0 (1.0–1.01)	0.057

*OR* odds ratio, *CI* confidence interval.

### Intraoperative monitoring (IOM) of stroke

Based on anesthesiology charts, we found no statistically significant hemodynamic parameters to predict or indicate the occurrence of intra-operative strokes (Table 3).

**Table 3:** Correlations between surgery-related infarcts and intraoperative parameters in intraoperative monitoring, awake surgery monitoring and anesthesiology parameters (univariate analysis).

IOM (n = 134)	No infarct (n = 118)	Infarct (n = 16)	Sig.
MEPs decline (%)	9 (7.6%)	2 (12.5%)	0.505

Awake monitoring (n = 73)	No infarct (n = 68)	Infarct (n = 5)	
In op language decline	9 (13%)	3 (60%)	**0.029** Fisher

Anesthesiology parameters (n = 234)	No infarct (n = 204)	Infarct (n = 30)	
ASA score ≥ 3, %	87 (42.6%)	13 (43.3%)	0.943
Anesthesia time ± SD, mins	267.30 ± 75.93	276.23 ± 67.69	0.543
MAP < 65 ± SD, mins	36.68 ± 44.43	40.73 ± 47.74	0.644
MAP < 65/ Anesthesia time ± SD, %	14.34 ± 18.30	15.40 ± 18.29	0.770
Baseline SBP ± SD, mmHg	134 ± 22	137 ± 30	0.505
Minimal SBP ± SD, mmHg	83 ± 16	79 ± 12	0.159
Minimal SBP/Baseline SBP Reduction ± SD, %	36.8 ± 14.2	33.9 ± 14.7	0.316
Minimal O_2_ ± SD, %	94.6 ± 3.9	95.6 ± 3.8	0.205

*IOM* intra-operative monitoring, *MEP* motor evoked potentials, *ASA* American Society of Anesthesiology score, *SBP* systolic blood pressure, *MAP* mean arterial pressure, *SD* standard deviation.

Intraoperative neurophysiology monitoring data were available in 134/239 cases. Two (18%) of the patients with IOM decline had infarcts on their postoperative MRIs, in comparison to 14 (11%) of those without decline (p = 0.505). Intraoperative neuropsychological monitoring findings in awake surgeries showed that intra-operative language decline was significantly more common among patients with infarcts (60%), in comparison to patients without infarcts (13%, p = 0.029, Table 3).

### Clinical and functional outcomes

Median overall survival of the study cohort was 12 months (95% CI 10.7–13.27). Among patients with stroke, median overall survival was 9 months (95% CI 5.43–12.57), as compared to 12 months (95% CI 10.61–13.39) among those without stroke (p = 0.053). Median overall survival among IDH-mutant WHO 4 astrocytoma (comprising 12% of the population) was significantly longer than IDH negative glioblastoma (23 vs. 12 months, p = 0.006), while patients with recurrent tumors (36% of the population) had significantly shorter survival from the day of operation in comparison to those with newly-diagnosed tumors (10 vs. 15 months, p < 0.001). Median progression free survival (PFS) of the entire cohort was 6 months (95% CI 4.92–7.09) with no significant differences between patients with and without stroke (p = 0.920).

Post-op median KPS and MRS were lower in the infarct group (p = 0.001), with no significant changes in these parameters up to 6 months after surgery (p = 0.160 and p = 0.146, respectively). Immediately after surgery, 9 of the patients with infarcts (30%) and 30 of those without (14.4%) experienced immediate new post-operative motor deficit or deterioration (p = 0.03). Six months after surgery, the rate of patients with motor deficits (either existing pre-op or occurring post-op) decreased from 57 to 37% in the infarcts group and from 37 to 25% in the non-infarcts group (p = 0.105 and 0.023, respectively). An overall comparison between the 2 groups over a half-year of post-op follow-up showed a higher rate of motor deficits among patients with infarcts (p = 0.003). New or deteriorating speech deficits were significantly higher in the immediate post-op period of dominant side surgery (n = 114) among patients with infarcts (n = 5/11, 46%), as compared to those without infarcts (n = 19/103, 18%, p = 0.037). Over 6 months of follow-up, the rate of patients with speech deficits (either existing pre-op or occurring post-op) improved significantly from 55 to 37% among patients without stroke (p = 0.006), while it decreased from 73 to only 64% (p = 0.638) among stroke patients. No difference was found in the rate of postoperative seizures among patients with or without strokes (Table 4).

**Table 4:** Clinical outcomes in 239 HGG patients with (n = 30) and without surgery-related infarcts (n = 209).

	KPS		MRS		Motor deficit (%)		Seizures (%)		Speech deficits (%)**	
	No infarct	Infarct	No infarct	Infarct	No infarct	Infarct	No infarct	Infarct	No infarct	Infarct
Pre-op	80 (30–100)	80 (40–90)	2 (0–5)	2 (1–5)	37%	47%	39%	47%	58%	73%
Immediate post-op					35%	57%	8%	3%	55%	73%
3 months post-op	90 (40–100)	70 (40–100)	2 (0–4)	3 (0–5)	26%	43%	8%	7%	43%	73%
6-months post-op	80 (30–100)	60 (20–100)	2 (0–5)	3 (0–5)	25%	37%	11%	17%	37%	64%
Sig.*	0.001 (Time = 0.160)		0.001 (Time 0.146)		0.003 (Time 0.056)		0.908 (Time 0.125)		0.05 (Time 0.453)	

*KPS* Karnofsky performance status, *MRS* modified rankin scale.

*Multivariate analysis for comparing infarct vs. non-infarct groups- Insular and temporal lesions.

**Speech deficits analysis was performed in a subgroup of dominant side surgeries (n = 114), comparing infarct (n = 11) vs. non-infarct groups (n = 103).

## Discussion

In this study we explored the incidence of intra-operative strokes following surgical resection of HGG, their risk factors, intra-operative monitoring and detection, as well as clinical outcomes.

### Stroke incidence and risk factors

The incidence of intra-operative stroke among patients who underwent surgical resection of HGG in our study was 12.5%. There is a wide variation in the rates reported in the literature, from 6.2 up to 80%, possibly due to the fact that many of the studies had combined populations of low and high grade tumors^6,7,10^. Our relatively homogenous population of HGG patients allowed us to look for potential predictors for surgery-related stroke.

We found 2 significant pre-operative risk factors for the occurrence of stroke, including tumor locations in the insula and temporal lobes. The insula is notorious for its high risk for morbidity due to adjacent eloquent landmarks and complex vascular supply, which is mainly based on frequent perforating arteries with no collateral flow. It is thought that brain ischemia during surgery results from direct vascular damage and coagulation, vasospasm, and kinking of arteries by retraction of the brain, which is often required in insular surgeries^10,12,15–17^. The current results go in line with our previous study in LGG patients, showing a higher risk for intra-operative stroke in insular surgeries, which constituted 11% of our cohort and nearly a quarter of those with infarcts^13^. A recent study by Strand et al. also found a higher risk for peri-tumoral strokes in the temporal lobes, yet they included both rim-pattern and sector shaped DWI restrictions in their definition of strokes^8^. Our study focused only on wedge-shaped ischemic lesions, while minimal or rim-pattern DWI changes in the periphery of the resection cavity, often defined as post-operative restrictive changes, were not considered as ischemic strokes^18^. We perform most of our surgical resections trans-cortically, yet our assumption is that even mild retraction, often required in temporal and insular tumors, might be enough to induce ischemia in diseased brain parenchyma, especially in recurrent or previously irradiated cases. In the case of trans-sylvian approach, the surgeon must be familiar with the regional anatomy and confident with the nuances of this technique in order to avoid direct damage to branches of the MCA. We use bipolar subcortically as needed, in order to coagulate bleeding tumor vessels. The use of bipolar in the vicinity of the lenticulostriate arteries should be cautious, as these vessels are particularly sensitive to thermocoagulation. Once the tumor itself is reached, we often complete the resection in a subpial approach, which is considered safer for the surrounding vasculature^15^. However, special attention should also be paid to vessels that may traverse the tumor, but actually supply critical normal parenchyma. Similar to previous reports^12^ and unlike others^10,13^, our current study showed no significant association between recurrent glioma surgeries and the risk for intra-operative stroke. From our findings, it seems that tumor location is more important than volume in predicting the risk for stroke, as pre-operative tumor volume did not show significant association with stroke. On the other hand, interestingly, infarct volume has been reported to correlate with pre-operative tumor volume^8^. Co-morbid conditions, such as history of diabetes mellitus, hypertension, ischemic heart disease and smoking did not show significant associations with surgery-related strokes. It seems that anatomical relationships between the tumor and adjacent blood vessels, as well as technical factors weigh more than systemic or general factors in determining the risk of intra-operative stroke.

### Intra-operative monitoring of stroke

Prompt detection of intra-operative stroke would allow immediate response and possibly the prevention of permanent neurological deficits. Possible intra-operative interventions in order to reduce tissue ischemia include releasing of retraction on brain parenchyma, irrigation with saline and local administration of papaverine and holding surgery^15^. So far, there has been no standardized tool which could consistently indicate the occurrence of stroke. In the current study we found that neuropsychological monitoring for language decline can indicate stroke during awake resections of HGG. These results are in line with our previous report, showing that confusion during awake craniotomy for resection of LGG is a significant indicator of intra-operative stroke^13,19^.

Intra-operative neuro-monitoring did not show significant associations with stroke in this study. These results are in line with our previous report in LGG surgeries where no significant association was found between neuro-monitoring changes and intra-operative stroke^13^. Previous studies in this topic have shown conflicting results. False-positive cases with IOM abnormality without post-operative deficits may occur when intra-operative measures are taken to correct the potential injury, such as adjusting retraction, raising blood pressure or halting surgery^16,17,20^. False-negative cases, where new surgery-related motor deficits occur without being detected by IOM abnormalities, can be explained in part by a “bypass” mechanism, in which Tc-MEP reach the pyramidal tract beyond the point of operation^20,21^. Transient post-operative motor deficits can also result by a delayed development of local edema around the pyramidal tracts or by resections involving the supplementary motor area, which often improve with time and do not necessarily manifest with IOM abnormalities^20–23^.

In addition, we did not find any anesthesiology and hemodynamic parameters to indicate or predict the occurrence of stroke, including factors that have been previously reported as significant, such as low MAP at the beginning of surgery and MAP decline during surgery. We aimed to keep systolic blood pressure below 120 mmHg both intra-operatively and during the post-operative recovery period in the neurosurgical intensive care unit.

#### Clinical outcomes

### Clinical and functional outcomes

Fourteen (47%) of the 30 patients with infarcts on postoperative imaging had new or deteriorating neurological deficits after surgery. The association between intra-operative stroke and surgically acquired immediate post-op deficits has been thoroughly studied^6,12^. Our study uniquely followed the evolution of the clinical manifestations of surgery-related stroke up to 6 months after surgery. In line with previous reports, there was a significantly higher rate of new or deteriorating motor deficits immediately after surgery among patients with infarcts (30% vs. 14.4%, p = 0.03). Interestingly, the rate of deficits decreased significantly over 6 months post-op among patients without infarcts, as opposed to those with infarcts (p = 0.023 and 0.105, respectively). Possibly, the majority of transient deficits among patients without infarcts were related to post-operative edema, while permanent ones were caused by direct motor or language tracts injuries. Glioma surgery-related strokes are mainly subcortical and deficits associated with this type of strokes are known to recover less than cortical ones^24^. However, these results are different from our previous report in LGG patients, which showed a significant improvement in the rate of motor deficits with time, even in those who sustained surgery-related strokes^13^. Possible explanations for these finding include the younger characteristic age of LGG patients, lack of exposure to adjuvant cancer treatments effects and their longer overall and progression free survival, which enabled them to go through rehabilitation and recovery, in comparison to HGG patients.

Similarly, among patients that had dominant side surgery, new or deteriorating immediate post-op speech deficits were significantly higher in patients with infarcts (46%), as compared to those without infarcts (18%, p = 0.037). All speech deficits correlated with the locations of either the superior longitudinal, inferior longitudinal or inferior fronto-occipital fasciculi. The rate of speech deficits that were not related to stroke decreased significantly over 6 months, unlike those that were stroke-related (p = 0.006 and p = 0.638, respectively). These neurological deficits were also linked to a significant decrease in both the KPS and MRS of stroke patients as compared to the rest of the study population (p = 0.001), which did not improve significantly over 6 months post-surgery^11^. In general, patients with symptomatic strokes, including either motor or speech deficits were referred to rehabilitation. As a result, in some cases, post-operative adjuvant treatment was delayed, yet in less severe symptomatic cases, adjuvant treatment was initiated in parallel to rehabilitation. We found no significant association between surgery-related stroke and median overall survival (p = 0.053). Our results are in line with a recent study by Lupa et al. which did not demonstrate an association between intra-operative stroke with neurological deterioration and survival in glioblastoma patients. Therefore, they concluded that aggressive resection in order to improve overall and progression-survival is warranted despite the potential risk for stroke^7^. However, it should be noted that important limitations to their project were the exclusion of patients with pre-op deficits, as well as cases complicated by small infarcts, which may also have critical impact on patients’ function and potentially survival, such as those involving the internal capsule^3,7,11^. It is noteworthy, however, that our results may have been influenced by the fact that 12% of our study population were IDH-mutant WHO grade 4 astrocytoma patients, who tend to have longer survival. Interestingly, none of the stroke patients were IDH-mutant, which may have also contributed to the shorter survival in this group^25,26^. We do not have a clear explanation for this finding and further studies should validate whether IDH-mutant tumors indeed tend to have less intra-operative strokes. Patients operated for recurrent tumors also showed significantly shorter survival from the day of operation, in comparison to newly-diagnosed tumors^27,28^, however, as mentioned above, we found no differences in the rates of recurrent operations between patients with and without surgery-related strokes. In summary, we infer that the occurrence of stroke may have a substantial impact on the patients’ neurological function and performance status, yet their effects on overall survival in the HGG patients’ population should be further studied. The risk for strokes, particularly in patients with temporal or insular tumors should be thoroughly discussed before surgery, including their interference with patients’ quality of life and their limited chances of recovery.

### Limitations

Due to the retrospective nature of our study we had to estimate and define the degree of the MRS and KPS functional scores. Furthermore, a larger study with more incidents of infarcts, could have better addressed critical questions, such as the ability to detect strokes by IOM and their impact on survival.

## Conclusions

Intraoperative ischemic events are more common in insular and temporal surgeries for resection of HGG. They can result in a lower performance status by impairing motor and speech functions. Further research is required in order to improve intra-operative detection and monitoring tools in order to prevent strokes or minimize their injury.

## Study population and methods

### Study population

We retrospectively studied risk factors for surgical ischemic complications in patients who underwent resection of HGG. We also evaluated their intra-operative monitoring data, and documented their short- and long-term implications. Between 2013 and 2017, 348 patients underwent either surgical resection or biopsy for HGG (either IDH wildtype glioblastoma or IDH-mutant WHO grade 4 astrocytoma) at our center. Of these, 239 patients underwent surgical resection of HGG with a full radiological and clinical dataset and comprised the study group. We excluded patients who only underwent biopsies (n = 39) and those with missing radiological (n = 19) and clinical data (n = 51), as certain patients did not continue follow-up at our institution. The most recent operation was considered as the index surgery in cases of patients who underwent more than one surgery during the study period. It is our common practice to use intra-operative navigation and 5-aminolevulinc acid as guiding tools during microsurgical resection of gliomas. The methods used for this study have been previously described by our group^13^.

### Clinical and demographic data

Hospital admission, surgical and discharge reports, including available documentation of clinical follow-up in the ambulatory or hospital setting up to 6 months post-op were reviewed for each patient. The following variables were documented: age, sex, body mass index, hand dominance, other malignancies, brain radiotherapy, cerebrovascular, cardiac and metabolic co-morbidities and recurrent tumors. Data on neurological manifestations, including motor and speech deficits and seizures that had been observed before and immediately (within hours) after surgery and at 3 and 6 months of follow-up were documented when available. Overall and progression free survival (PFS), as well as KPS and Modified Rankin Score (MRS) were also documented.

### Radiological data

**Figure 1:**
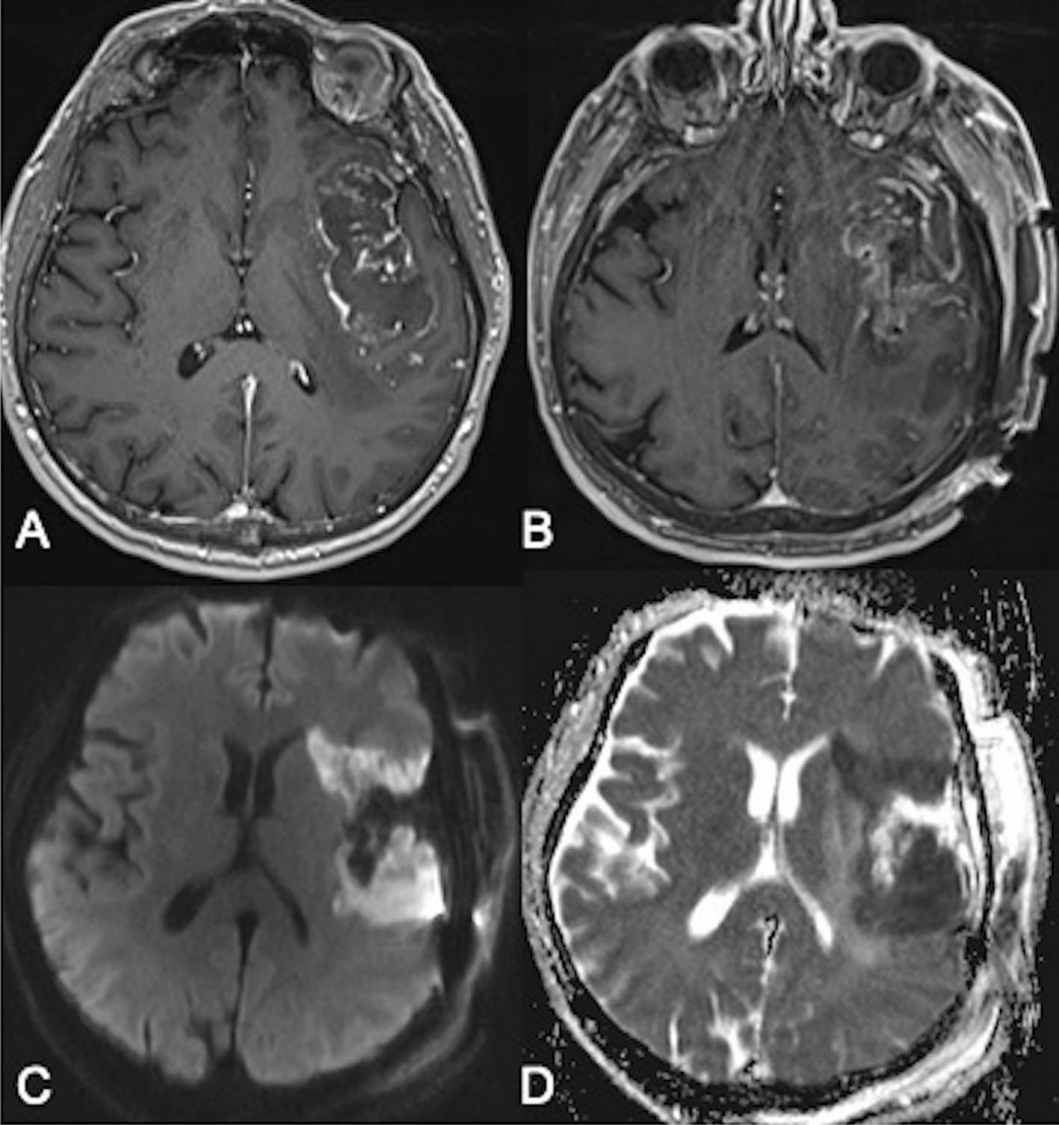
T1-weighted MRI with contrast of a 64 year-old male with an insular glioblastoma before (**A**) and after (**B**) surgery. Post-operative diffusion-weighted imaging (**C**) and apparent diffusion coefficient (**D**), showing a large wedge-shaped surgery-related infarct.

We documented tumor volume and location, tumor enhancement, and extent of resection (EOR) calculated from pre- and immediate postoperative magnetic resonance imaging (MRI) studies. The EOR was calculated using the following formula: (preoperative − postoperative tumor volume)/preoperative tumor volume × 100). The volume of blood products rather than the volume of the residual tumor was confirmed by comparing T1-weighted gadolinium-enhanced and non-enhanced MRIs. FLAIR sequence was used for measuring the non-enhancing component of the tumors. Surgery-related arterial brain infarcts were diagnosed based on immediate (within 48 h) postoperative MRIs and the presence of wedge-shaped restrictive changes on diffusion-weighted imaging (DWI) and apparent diffusion coefficient techniques (ADC), adjacent to the resection cavity (Fig. 1). Minimal DWI changes in the periphery of the resection cavity, often defined as post-operative restrictive changes, were not considered as ischemic strokes. MRI reports were provided by neuroradiologists blinded to clinical outcomes.

### Intraoperative neurophysiologic and anesthetics data

Recordings of transcranial, direct cortical and subcortical motor evoked potentials of IOM were analyzed, as previously reported by our team^12–14^. Monitoring during awake procedures was done by a trained neuropsychologist and included language assessments for the detection of semantic, phonemic, and expressive decline, comprehension, and confusion. Anesthesiology data included preoperative American Society of Anesthesiology (ASA) score, anesthesia duration, blood pressure measurements before and during surgery, and oxygen saturation.

### Statistical analysis

Survival times were analyzed based on the Kaplan–Meier product limit method. Average values were presented as mean ± standard deviation or median and range, and a *P*-value of less than 0.05 (two-sided) was considered statistically significant. Characteristics between groups and subgroups of categorical data were compared using the Pearson’s χ^2^ test and Fisher's exact test. Multivariate logistic regression analysis was used to evaluate risk factors for developing intraoperative stroke. Repetitively measured variables were analyzed using generalized estimating equations. Missing cases were not included in the analysis. Statistics were performed using SPSS 21.0 software (SPSS Inc, Chicago, IL).

### Ethical approval

This study was approved by the Tel-Aviv Sourasky Medical Center institutional ethics committee, reference number: 0768-17-TLV. The study was performed in accordance with the relevant guidelines and regulations.

### Consent to participate

The ethics committee of the Tel-Aviv Sourasky Medical Center waived the requirement of informed consent due to the retrospective nature of the study.

## Data Availability

The datasets generated during and/or analyzed during the current study are available from the corresponding author on reasonable request. In the future we may consider asking our patients for their permission to share clinical data for research puropses.
